# Does the Sphingosine 1-Phosphate Receptor-1 Provide a Better or Worse Prognostic Outcome for Breast Cancer Patients?

**DOI:** 10.3389/fonc.2018.00417

**Published:** 2018-09-27

**Authors:** Nigel J. Pyne, Susan Pyne

**Affiliations:** Strathclyde Institute of Pharmacy and Biomedical Sciences, University of Strathclyde, Glasgow, United Kingdom

**Keywords:** Sphingosine 1-phosphate, breast cancer, Sphingosine 1-phosphate receptor, sphingosine kinase, disease specific survival, cell growth, cell migration

## Introduction

The bioactive lipid, sphingosine 1-phosphate (S1P) is formed by the sphingosine kinase-catalyzed phosphorylation of sphingosine. There are two isoforms of sphingosine kinase termed SphK1 and SphK2 that are encoded by different genes and which exhibit distinct sub-cellular localization and biochemical properties to regulate overlapping and non-overlapping biology (1). S1P is either degraded by S1P lyase to produce (*E*)-2-hexadecenal and phosphoethanolamine or reversibly dephosphorylated by S1P phosphatase to regenerate sphingosine (1). S1P can be exported from cells [through transporter proteins such as Spns2 (2), MFsd2b (3), and some ABC proteins (4)] to bind to a family of five S1P-specific G protein coupled receptors (S1P_1−5_) (5). There is substantial evidence to suggest that S1P has a critical role in cancer. For example, the knockout of lymphatic endothelial *Spns2* reduces pulmonary metastasis via a mechanism that involves induction of a lymphopenia and an increase in effector T cells and natural killer (NK) cells to enhance tumor cell killing in the lung (6). Other evidence supports a role for S1P in transformation, epithelial mesenchymal transition (EMT), invasiveness, cancer cell survival and replicative immortality, tumor neovascularisation, and the Warburg effect (7).

## Role of S1P in defining breast cancer prognosis

Lei et al. (8) used data mining and the Kaplan-Meier plotter (http://kmplot.com/analysis/index.php?p=service&cancer=breast) to establish relationships between messenger RNA levels (rather than protein) of SphK1, SphK2, sphingosine 1-phosphate receptor 1 (S1P_1_), or sphingosine 1-phosphate receptor 2 (S1P_2_) and 10 year relapse free survival of non-classified breast cancer (BCA) patients. High SphK1 mRNA in tumors had no effect on 10 year relapse free disease survival, while high SphK2 mRNA was found to have a positive impact in patients with non-classified, or basal type BCA and those who had received adjuvant therapy. Lei et al. (8) also reported that high S1P_1_ mRNA in tumors was associated with improved survival in patients with non-classified BCA and in those receiving treatment, but had no effect on patients with basal cell type BCA. In addition, improved survival was associated with high S1P_2_ mRNA in tumors from patients with non-classified BCA, basal cell type BCA and those patients receiving treatment.

We suggest that the interpretation of these findings should be viewed with considerable caution. In this regard, Kaplan-Meier plot analysis relating gene expression, i.e., mRNA transcript, with prognosis does not consider the possibility that mRNA transcript expression may not reflect specific protein levels. Indeed, it is the protein itself that performs the signaling function, and not the mRNA. In this regard, if the turnover of a particular protein is slow, its mRNA levels might be low. Therefore, a reciprocal relationship could exist between mRNA and protein levels. In this case, interpretation of the results by Lei et al. (8) would be completely reversed. For instance, it could be concluded that high expression of S1P_1_ protein (low mRNA transcript) in BCA tumors is linked with a poorer prognosis. Indeed, this was precisely the findings of Watson et al. (9) where the estrogen receptor positive (ER^+^) breast cancer tumors of a population of 304 patients was analyzed. In this respect, high protein expression of S1P_1_ or SphK1 (detected using immunohistochemical staining with anti-S1P_1_ antibody or anti-SphK1 antibody) in tumors was associated with *earlier* disease recurrence and *reduced* disease specific survival times. The difference for S1P_1_ is not trivial with mean disease recurrence and disease specific survival times being shortened by between 3 and 8 years, respectively.

In addition, a relationship with poor prognosis was evident for both membrane and cytoplasmic localized S1P_1_; the latter probably reflects receptors that have been endocytosed in response to S1P, and which are capable of persistent signaling (10). In contrast, combined high nuclear localized S1P_2_ and c-Src protein expression in tumors is associated with increased disease specific survival times (11). Indeed, a thorough analysis of the impact of S1P receptor sub-types on breast cancer prognosis should also consider sub-cellular localization and this can only be performed using analysis of protein with validated anti-S1P receptor antibodies. There is a very substantial body of evidence that demonstrates that S1P_1_ promotes tumorigenesis through, for example, enhanced cell survival, migration, and angiogensis (7). The role of S1P_2_ in cancer is more controversial with evidence that it can either promote or inhibit tumorigenesis (12, 13)^.^ To rationalize these findings, we have proposed that the positive and negative function of the S1P_2_ receptor in cancer might be dependent on its sub-cellular localization (14).

Patient stratification based on ER, progesterone receptor and HER2 status is also important. This is exemplified by the finding that high expression of SphK1 protein in tumors actually increases disease specific survival times in ER^+^/HER2^+^ patients. Indeed, high expression of SphK1 induces a desensitization/tolerance to HER2-mediated signaling in ER^+^/HER2^+^ breast cancer cells (15).

## S1P receptor-dependent mechanisms

To provide a mechanistic explanation for the clinical findings, Lei et al. (8) reported the inhibition of colony formation by MCF-7 cells (ER^+^) or triple negative MDA-MB-231 cells in response to treatment with S1P (4–10 μM) for 2 weeks. Using shRNA knockdown of either S1P_1_ or S1P_2_ in MCF-7 and MDA-MB-231 cells, the authors showed that the inhibitory effect of S1P on colony formation was enhanced in both cell types upon S1P_2_ knockdown whereas S1P_1_ knockdown reduced the effect in MDA-MB-231 cells and was without effect in MCF-7 cells. However, there was no evidence presented to indicate that the effect of S1P on colony formation actually reflects an inhibition of growth assessed by, for instance, cell-cycle analysis. This is critically important in order to validate the conclusions, because S1P stimulates migration via S1P_3_ (15) and S1P_1_ (16). In contrast, the S1P_2_ receptor has been linked with inhibition of cell migration (17). Therefore, the findings by Lei et al. (8) regarding the effect of shRNA knockdown of S1P_2_ are in fact consistent with a relief of the inhibitory constraint of S1P_2_ on the migration of MCF-7 and MDA-MB-231 cells in response to added S1P. This would reduce colony formation by enhancing dispersal and thereby preventing colony residency. The knockdown of S1P_1_ in reducing the inhibitory effect of S1P on colony formation of MDA-MB-231 cells is consistent with ablation of the positive effect of S1P_1_ on migration, which would enhance colony formation/residency. Thus, colony formation (without more extensive cell cycle analysis and assessment of migration) does not provide confirmation that S1P inhibits tumorigenesis *in vitro*. The effect of treatment with S1P for 2 weeks on S1P receptor expression was also not examined by Lei et al. (8).

The major functional S1P receptor type in MCF-7 cells is, in fact, the S1P_3_ receptor which induces activation of the ERK-1/2 pathway via transactivation of the EGF receptor to promote migration (18, 19). S1P_3_ expression/function was not analyzed by Lei et al. (8). Furthermore, the EGF receptor kinase is positively linked with tumorigenesis and this is a rationale for the use of EGF receptor kinase inhibitors for the treatment of cancer. Therefore, in contrast to the authors' conclusions, these prior published findings strongly implicate S1P in promoting breast cancer tumorigenesis *via* an EGF receptor-dependent mechanism. Finally, the study by Lei et al. (8) takes no account of the other responses that S1P regulates through its receptors and which are of relevance to cancer, such as migration, angiogenesis and chemo-resistance.

## Conclusion

We conclude that meaningful analysis of the impact of tumor S1P receptor sub-type expression on the survival of breast cancer patients requires analysis of actual protein levels and not mRNA. There is also a need to stratify patient cohorts to consider for instance, ER and HER2 expression. In addition, the effects of S1P on colony formation of MCF-7 and MDA-MB-231 cells requires analysis of cell cycle progression and migration with respect to the function of different S1P receptor sub-types. Moreover, a comprehensive analysis of the protein expression of all S1P receptors in the breast cancer cells studied and their individual biological functions should be interrogated. This is important as it is unknown whether, for instance, knockdown of S1P_1_ and/or S1P_2_ has an effect on the expression of the other S1P receptors and whether there is redundancy and/or divergent biological functions of these receptors. Indeed, this appears to be the case as evidenced by lack of effect of S1P_1_ knockdown in MCF7 cells, where S1P_3_ may play a more prominent role. Finally, Lei et al. (8) conclude that breast cancer could be treated with S1P_1_ specific agonists, but provide no *in vivo* data to support this. In contrast, we argue that the weight of published evidence clearly suggests that such S1P_1_ specific agonists have the potential to reduce disease specific survival times for breast cancer patients which is counterproductive in terms of therapy.

## Author contributions

All authors listed have made a substantial, direct and intellectual contribution to the work, and approved it for publication.

### Conflict of interest statement

The authors declare that the research was conducted in the absence of any commercial or financial relationships that could be construed as a potential conflict of interest.
